# COVID-19 and persons with disabilities in the Philippines: A policy analysis

**DOI:** 10.34172/hpp.2021.38

**Published:** 2021-08-18

**Authors:** Jacqueline Veronica Velasco, Joseph Christian Obnial, Adriel Pastrana, Hillary Kay Ang, Paulene Miriel Viacrusis, Don Eliseo Lucero-Prisno III

**Affiliations:** ^1^Faculty of Medicine and Surgery, University of Santo Tomas, Sampaloc, Manila, Philippines; ^2^Faculty of Management and Development Studies, University of the Philippines Open University, Los Banos, Laguna, Philippines; ^3^London School of Hygiene and Tropical Medicine, London, United Kingdom

**Keywords:** COVID-19, Disabled persons, Philippines, Policy making

## Abstract

**Background:** The coronavirus disease 2019 (COVID-19) pandemic warrants an immediate response. Persons with disabilities (PWDs) are one of the most vulnerable populations susceptible to marginalization. While there are existing guidelines in the Philippines that aim to assist their basic needs, a call for inclusivity in policymaking for the COVID-19 response is highly advocated. This paper aims to analyze existing policy guidelines concerning the welfare of PWDs in the country based on several policy domains.

**Methods:** Relevant documents were acquired through extensive search of government and nongovernment websites and news agencies. Literature included memorandums, circulars, and news bulletins in the period between January 2020 to May 2021. This study conducted a framework analysis on policies enacted by the Philippine government during the COVID-19 pandemic concerning PWDs. The framework was divided into eight areas: access to (1) information, (2)healthcare, (3) education, and (4) financial support, (5) protection from infection in residential settings, (6) reasonable accommodation, (7) consideration for disabled people facing multiple exclusions, and (8) inclusion to decision-making process.

**Results:** Fifteen PWD related COVID-19 response documents from the Philippines were reviewed and analyzed. Most policies corresponded to themes related to financial support and reasonable accommodations. Most documents were limited to proposals and suggestions with only a few documents including specific details on how the program will be implemented and monitored.

**Conclusion:** The state has proven its cognizance for PWDs; however, implementation and its impact remain to be seen. The government needs to evaluate these efforts to identify gaps and barriers. A comprehensive national database should be implemented to centralize registration of PWDs, and efforts should be made to inform and educate PWDs of their rights and of existing programs. Most importantly, PWDs should be included in the discourse and decision-making process to ensure programs are acceptable and accessible.

## Introduction

The ongoing coronavirus disease 2019 (COVID-19) pandemic has significantly affected the lives of many. One population group highly impacted are persons with disabilities (PWDs). They are disproportionately affected not only because of their already heightened vulnerability to the disease, but also due to the regulations implemented to control the spread of COVID-19.^1^ Worldwide, approximately over 1 billion people or about 15% of the world’s population are reported to be living with some form of disability. In the Philippines, approximately 1.44 million or 1.57% of its population have a certain disability.^2^ The Philippines is currently second among the Southeast Asian regions in terms of COVID-19 infections, which logged a total of 1 240 716 active cases as of June 3, 2021.^3^

Even before the pandemic, PWDs have remained one of the most vulnerable and most unheard voices in our society.^4^ They experience inequality, violation of dignity, and deprivation of autonomy,^5^ which is only expected to worsen because of the global health crisis. United Nations Special Rapporteur, Catalina Devandas, stated that persons with disabilities are not provided with the adequate support and protection they need with the ongoing pandemic.^6^

The regulations put in place in response to the pandemic produced multiple problems to its population. Most of the PWDs are more likely to be unemployed or working in the informal sector, making them financially insecure, and further increasing the burden on this population group.^1^

Stakeholders, such as the government and leaders of humanitarian sectors, are called to actively participate in the empowerment of PWDs by addressing the barriers they may face with COVID-19.^7^ International guidelines have already been established in response to the call for disability inclusion. Because of the diversity of disabilities, it is encouraged to collect information from various groups of people through means completely devoid of discrimination or exclusion. In return, public information, especially regarding health and safety measures, must also be translated into accessible formats through accessible technology^7,8^ Aside from information accessibility, persons with disabilities must be ensured with proper medical and income security.^8,9^ The International Labor Organization emphasized that both mainstream and targeted measures must be taken to ensure social protection during COVID-19 lockdowns.^7,10^ It is recommended that significant funding and support be provided to non-government organizations, especially those concerned with humanitarian responses to maximize their operational capacity and mobilization.^9^

Everyone must understand that inclusivity is critical in forming guidelines that would fortify the nation’s COVID-19 response. Providing data analysis to the existing policies may alleviate the lack of studies promoting PWD inclusivity in the Philippines and provide awareness of the gaps in the national COVID-19 response strategies for PWDs.

In assessing the current policies and guidelines, this paper aims to identify the existing gaps and barriers of PWD inclusivity in the Philippines, ensure disability-inclusive COVID-19 response and recovery, and promote PWD inclusivity in the future policy-making processes of the country. We had the objective of analyzing existing policies regarding PWDs during the COVID 19 pandemic in the Philippines. We will categorize the existing guidelines according to each policy domain and provide updates on the ongoing progress of the approved policies by the designated government organizations.

## Materials and Methods

Relevant documents were retrieved through extensive search of government websites and news agencies. Google search engine and Google Scholar were also used. Included data came from peer-reviewed journals, gray literature, and reports. Gray literature included memorandums, circulars, and news bulletins from the different branches of the Philippine government and national and international advocacy organizations and was restricted to the period between January 2020 to May 2021 to focus on the efforts made during the COVID-19 outbreak. Key search terms used were persons with disabilities, Philippine government, COVID-19, policy, and government agencies (e.g., Department of Labor and Employment, Department of Interior and Local Government, etc.).

Documents were assessed and analyzed employing framework analysis based on the framework created by Sakellariou et al (Table 1),^11^ which used documents from the WHO, United Nations and the Economic Commission for Latin America and the Caribbean. The applicability of the framework to the Southeast Asian setting was assessed using regional guidelines from the WHO and organizations such as the International Labor Organization and was found to be applicable and comprehensive enough without need for further modification.

**Table 1:** Thematic framework adopted from the work of Sakellariou et al^11^

**Area**	**Explanation**
Accessible information	Provision of all information in accessible formats, including sign language translation, Braille script and easy read.
Access to healthcare	Removal of financial barriers to care, and measures taken to ensure equitable access to healthcare, including measures addressing disability-based discrimination.
Access to education	Measures taken to ensure remote learning is fully accessible.
Financial support	Provision of financial support (e.g., cash transfers or benefits) to disabled people and their family members, if they had to stop working, and measures taken to ensure access to financial support, including automatic extension of disability benefits.
Protection of people living in residential settings	Measures taken to ensure people living in residential care are protected from infection.
Reasonable accommodations for disabled people	Adjustments to public health measures to accommodate the needs of disabled people, including flexibility in restrictions on movement in public spaces.
Consideration for disabled people facing multiple exclusions	Measures taken to protect disabled people who are at increased risk of social exclusion and poverty, such as women, children, homeless people, and prisoners.
Inclusion to decision making process	Inclusion of disabled people and their representative organizations to advisor and decision-making bodies.

## Results

A total of 15 PWD related COVID-19 response documents (Table 2) from the Philippines were considered for analysis. These documents were reviewed and analyzed within a framework policy analysis. Researchers found policies that corresponded to all 8 themes as plotted on Table 3. The policies and programs were created for the protection of the rights of PWDs under emergency circumstances. Most documents were limited to proposals and suggestions, leaving the implementing agencies to come up with their own plans for execution. Only a few documents included specific details on how the program will be implemented and monitored.

**Table 2:** PWD related COVID-19 response documents

**No.**	**Document**	**Source**
1	House Resolution No. 955	House Resolution No. 955. (2020). Accessed at https://congress.gov.ph/legisdocs/basic_18/HR00955.pdf
2	DOH: Philippines Covid-19 Emergency Response Project Environmental and Social Management Framework	Department of Health. Philippines Covid-19 emergency response project environmental and social management framework. November 2020. Available from: https://doh.gov.ph/sites/default/files/basic-page/ESMF%2011252020.pdf. Accessed 4 April 2021.
3	CHR: Human Rights advisory series on human rights in the time of Covid-19 in the Philippines in pursuit of the rights-based model of disability amid the COVID-19 pandemic in the Philippines CHR (V) A2020-009	Commission on Human Rights. CHR (V) A2020-009. Human Rights Advisory in the Time of Covid-19 in the Philippines in Pursuit of the Rights-Based Model of Disability Amid the Covid-19 Pandemic in the Philippines. Available from: http://chr.gov.ph/wp-content/uploads/2020/05/Human-Rights-Advisory-HR-in-the-times-of-COVID-19-in-the-PHL-in-pursuit-of-the-rights-based-model-of-disability-amid-the-COVId-19-pandemic-the-in-the-PHL-CHR-V-A2020-009.pdf. Accessed 4 April 2021.
4	DOLE: PWDs get livelihood aid amid pandemic	Department of Labor and Employment (DOLE). PWDs get livelihood aid amid pandemic. September 2020. Available from: https://www.dole.gov.ph/news/pwds-get-livelihood-aid-amid-pandemic/. Accessed 4 April 2021.
5	DOH on deployment of vaccines: The Philippine National Deployment and Vaccination Plan for COVID-19 vaccines	Department of Health (DOH). The Philippine national deployment and vaccination plan for Covid-19 vaccines. January 2021. Available from: https://doh.gov.ph/sites/default/files/basic-page/The%20Philippine%20National%20COVID-19%20Vaccination%20Deployment%20Plan.pdf. Accessed 4 April 2021.
6	DSWD: Memorandum Circular No.09 Series of 2020 Omnibus guidelines in the implementation of the emergency subsidy program of the department of social welfare and development	Department of Social Welfare and Development (DSWD). Memorandum Circular No. 09 - Omnibus Guidelines in the Implementation of the Emergency Subsidy Program of the Department of Social Welfare and Development. Available from: https://www.ncda.gov.ph/wp-content/uploads/2020/04/MC-09-s2020-OMNIBUS-GUIDELINES-EMERG-SUBSIDY-PROG-DSWD.pdf. Accessed 4 April 2021.
7	Republic of the Philippines Inter-Agency Task Force for the management of emerging infectious diseases Omnibus guidelines on the implementation of community quarantine in the Philippines with amendments as of June 3, 2020	Inter-Agency Task Force (IATF). Omnibus Guidelines On The Implementation Of Community Quarantine In The Philippines. Available from: https://www.officialgazette.gov.ph/downloads/2020/06jun/20200603-omnibus-guidelines-on-the-implementation-of-community-quarantine-in-the-philippines.pdf. Accessed 4 April 2021.
8	PhilHealth guarantees continuing coverage for COVID-19 patients	PhilHealth. PhilHealth guarantees continuing coverage for Covid-19 patients. April 2020. Available from: https://www.philhealth.gov.ph/news/2020/cont_coverage.php#gsc.tab=0. Accessed 4 April 2021.
9	Administrative Oder 39 Authorizing the grant of a one time financial assistance of P20,000 to employees’ compensation pensioners	Administrative Order No. 39. (2021). Authorizing the Grant of a One-Time Financial Assistance of P20,000.00 to Employees’ Compensation Pensioners. April 2021. Available from: https://pcoo.gov.ph/news_releases/administrative-order-no-39-authorizing-the-grant-of-a-one-time-financial-assistance-of-p20000-00-to-employees-compensation-pensioners/#:~:text=Administrative%20Order%20No.,Pensioners%20%E2%80%93%20Presidential%20Communications%20Operations%20Office. Accessed: 5 April 2021.
10	DTI to launch Biz-Ability online trade fair for Persons with disability	Department of Trade and Industry (DTI). DTI to launch Biz-Ability Online Trade Fair for Persons with Disability. July 2020. Available from: https://www.dti.gov.ph/archives/news-archives/dti-biz-ability-online-trade-fair-pwd/?fbclid=IwAR070KEDAcL94XGf4oxGtw4Bl-midnH4IebkLRAyPdrOheA-oEy7AtW_vEo. Accessed: 4 April 2021.
11	DSWD advises seniors, PWDs to send representatives to claim SAP	Department of Social Welfare and Development (DSWD). DSWD advises seniors, PWDs to send representatives to claim SAP. May 2020. Available from: https://www.dswd.gov.ph/dswd-advises-seniors-pwds-to-send-representatives-to-claim-sap/. Accessed 5 April 2021.
12	Office of the President of the Philippines: Memorandum from the Executive Secretary Additional measures to address the rising cases of COVID-19 in the country (March 21, 2020)	Office of the President of the Philippines: Memorandum from the Executive Secretary. Additional Measures to Address the Rising Cases of COVID-19 in the Country. March 2021. Available from: https://www.officialgazette.gov.ph/downloads/2021/03mar/20210321-MEMORANDUM-FROM-THE-ES-RRD.pdf. Accessed 5 April 2021.
13	Guidelines on providing proper welfare of persons with disabilities during the enhanced community quarantine due to the coronavirus 2019 (Covid-19) pandemic	Department of Interior and Local Government (DILG). Memorandum Circular No. 2020-066 - Guidelines on Providing Proper Welfare of Persons with Disabilities during the Enhanced Community Quarantine due to the Corona Virus 2019 (Covid-19) Pandemic. March 2020. Available from: https://www.ncda.gov.ph/wp-content/uploads/2020/04/DILG-MC-066-s-2020-Guidelines-on-the-Providing-Proper-Welfare-of-PWDs-during-ECQ.pdf. Accessed 5 April 2021.
14	Client Update: Philippines 2020 June The Omnibus Guidelines on community quarantine in the Philippines	Rajah & Tann Asia. Client Update: Philippines - The Omnibus Guidelines on Community Quarantine in the Philippines. June 2020. Available from: https://ph.rajahtannasia.com/media/3941/iatf-revised-omnibus-guidelines-as-of-22-may-2020-client-updates-rt-jun-2-2020-002.pdf. Accessed 5 April 2021.
15	Department of Labor and Employment 15 July 2020 Moving towards disability-inclusive recovery in employment and livelihood in the time of COVID-19	Department of Labor and Employment (DOLE). Moving towards disability-inclusive recovery in employment and livelihood in the time of COVID-19. July 2020. Available from: https://www.dole.gov.ph/news/moving-towards-disability-inclusive-recovery-in-employment-and-livelihood-in-the-time-of-covid-19/. Accessed 4 April 2021.

**Table 3:** Government responses to COVID-19 based on the thematic framework

**Area**	**Policies implemented**
Accessible information	Requirement to have Filipino sign language interpreters on broadcast media and community-based trainings in local health centers [1, 2] Accessible information in integration and preparedness activities [2] Published recommendations to make information and communication accessible to all PWDs especially children with disabilities [3]
Access to healthcare	Law in mandatory coverage under Philippine Health Insurance remains in full force [3] Sickness and disability benefits, medical reimbursements, free physical and occupational therapy for PWD beneficiaries of KaGabay Program [4] Department of Health’s Vaccination Program includes PWD under category B [5]
Access to education	Free skills and entrepreneurial training for PWD beneficiaries of KaGabay Program [4]
Financial support	Health insurance coverage for COVID-19 patients such as the use of PhilHealth [3] Providing employment opportunities in the public and private sector [3] Value added tax exemptions on the sale of goods and services [3] Livelihood packages were distributed to PWD beneficiaries of KaGabay Program [4] Cash assistance for PWD beneficiaries [4, 6, 7] Livelihood assistance to vulnerable groups [7] Livelihood assistance to vulnerable groups [7] PWDs are entitled to discounts for health services and treatments [8] Financial assistance of P20 000.00 to employees’ compensation pensioners [9] Online Trade Fair for our micro, small, and medium enterprises owned by persons with disability [10]
Protection of people living in residential	Ensure and monitor minimum public health standards adherence at home by distribution of face masks and face shields to the vulnerable sectors [7] Prohibition of all mass gatherings [7] Prioritization in distribution of food pack, medicine and vitamins while under enhanced community quarantine [13]
Reasonable accommodations for disabled people	Allocation of priority lanes to PWDs [3] Penalizing acts that tend to vilify PWDs [3] PWDs can send a representative to claim their emergency subsidy [11] Standby ambulance and paramedics at subsidy payout sites [11] Door to door delivery of cash aid to PWDs who are heads of the family [11] PWDs may engage in outdoor exercises and non-contact sports as part of their physical therapy [12] Transportation services are to be readily available to the barangay captains upon the request of persons with disability [13] Inclusion of PWDs in the list of beneficiaries in distribution of food pack and food items received including medicine and vitamins [13]
Consideration for disabled people facing multiple exclusions	The Regional Federation of PWDs act as a referral unit to help PWDs access their needs and services during the quarantine period. [4] Permitted at home therapy services for PWDs [7,14] Determination of the needs of PWDS in labor and employment via survey [15]
Inclusion to decision making process	Recommendation for PWDs to be included in decision making processes current concerns on the accessibility, acceptability and quality criteria and in emergency response and relief operations. [3]

References found in Table 2.

### Accessible information

The Emergency Social Management Framework (ESMF) and congressional resolutions required Filipino sign language interpreters on broadcast media and community-based training of Filipino sign language to facilitate information dissemination regarding COVID-19 screening and quarantine facilities. ESMF’s integration of information for preparedness activities were also given importance to provide accessible formats such as print materials in Braille or large print, sign language interpretation, captions, audio provision, and graphics for PWDs.

### Access to healthcare

The government, especially the Department of Health, provided measures to include PWDs in their access to healthcare. Sickness and disability benefits, medical reimbursements, and free physical and occupational therapy for PWD beneficiaries of KaGabay Program were provided by the Employees Compensation Commission (ECC). Furthermore, healthcare inclusivity was covered in full force by the Philippine Health Insurance Corporation (PhilHealth). This is seen in the deployment of the vaccination program in the Philippines which included PWDs under group B, along with frontline workers and the indigenous population. Under this priority group, PWDs will be the second group prioritized after Group A which includes frontline health workers, senior citizens, and those with comorbidities.

### Access to education

In response to education access, DOLE’s Employees Compensation Program provides PWD beneficiaries with free skills and entrepreneurial training. Although these were not expounded elaborately, these will be of importance to the PWDs due to the unpredictable economic changes brought about by the COVID-19 pandemic.

### Financial support

Regarding financial support, PWDs are entitled to receive employment opportunities, livelihood packages, and monetary benefits through different programs. The ECC together with IATF has agreed to give out cash and livelihood assistance to PWDs. Through Administrative Order 39 which follows Article 177 of Presidential Decree No. 626 (s. 1074), entitled “Further Amending Certain Articles of Presidential Decree No. 442”, 20 000 Pesos (420 USD) were given to pensioners of the employees’ compensation program including PWDs under this. For venturing entrepreneurial PWDs, Biz-Ability Online Trade Fair (online trade fair) was made for PWDs to be able to safely sell their products. Discounts for health services and treatments for their disability were provided to PWDs through PhilHealth.

### Protection of people living in residential settings

Community Quarantines were implemented to protect people in their residential settings from COVID-19 infections. This entails restriction of mass gatherings and only allowing travel for essential needs. Vulnerable groups such as ages 18 and below, seniors and other vulnerable groups including PWDs have stricter quarantine measures. They are not allowed to enter public spaces unless absolutely necessary. Government groups such as the IATF also distributed face masks and face shields to vulnerable groups such as PWDs for further protection.

### Reasonable accommodations for disabled people

New policies were implemented to adapt to the needs of PWDs amidst the COVID-19 pandemic. These include recommendations to allocate priority lanes and penalize acts that vilify PWDs. PWDs were allowed to send representatives to claim their emergency subsidies. Cash assistance was also delivered door-to-door especially to PWDs who are heads of their family. Standby ambulances and paramedics at subsidy payout sites were provided. Continuation of physical therapy services were also allowed for PWDs. PWDs were allowed to go outside their residential homes as part of their exercise regimen in therapy upon presentation of a PWD ID or prescription from the physician. Apart from these, PWDs were also allowed to request transportation help from their local government unit should they need it.

### Consideration for disabled people facing multiple exclusions

In an effort to recover from the effects of COVID-19 pandemic the government created a referral unit, the Regional Federation of PWD, to help PWDs access their needs and services. Surveys were also deployed to determine the needs of PWDs in labor and employment.

### Inclusion to decision making process

Only one document recommended the inclusion of PWDs in the decision-making processes in their concerns about policies about accessibility, acceptability, and quality criteria and in emergency response. However, it remains to be seen if this was properly implemented.

## Discussion

PWDs continue to belong to the most vulnerable populations. Despite increasing calls for inclusivity, they still face several health and financial challenges.^12^ To make matters worse, most of them are poor, finished lower levels of education, and face discrimination and difficulty finding employment.^13^ The global health crisis has placed them in an even more precarious situation. They are left unprotected from COVID-19 related health issues on top of trying to provide for their needs.

In March 2020, as COVID-19 cases started to rise in the Philippines, the initial response of the government was to impose quarantine measures of varying severity. Under the highest classification of community quarantine, the general population was ordered to stay home. Travel was limited to accessing essential goods and services, and most forms of public transportation were suspended. These restrictions loosened as the area’s quarantine classification decreased. However, even under the lowest classification, those under the age of 15 or over the age of 65, pregnant women, and those with immunodeficiency, comorbidities or other health risks were still prohibited from traveling outside of their residences unless absolutely necessary.^14^ Because of these restrictions, the economy was greatly halted and millions of Filipinos, including PWDs, were left jobless. In a rapid assessment survey, 70% of PWDs reported that their employment had been affected by the pandemic, ranging from a shift to work from home to being at risk of losing their jobs.^15^ Consequently, PWDs and other marginalized groups face increasing fears due to uncertainty magnified by COVID-19.

The Philippine government recognized these issues and enacted policies and programs to account for the needs of PWDs prior to and during the pandemic. Examples of these include the Magna Carta of Disabled Persons’ and the Filipino Sign Language Act. The Republic Act (R.A.) No. 7277 also known as the Magna Carta for Disabled Persons’ grants rights and privileges for disabled persons. Disabled persons under this act are defined as those suffering from restriction or different abilities, because of a mental, physical, or sensory impairment, to perform an activity in the manner or within the range considered normal for a human being. Through this act, disabled persons are granted rights under the following domains: employment, education, health, auxiliary social services, telecommunications, accessibility, and political and civil rights. It also prohibits discrimination against disabled persons on employment, transportation, and on use of public accommodation and services.^16^ In line with this, is the enactment of R.A. No. 11106, known as the Filipino Sign Language Act. It mandates the use of Filipino sign language in broadcast media and government transactions among other situations.^17^ The response of the Philippine government to the increased and novel needs of the PWDs during this pandemic has been guided by these provisions, as evidenced by the COVID-19 responses that fall under the themes of the framework. Unfortunately, there are gaps in implementation of these acts that have been highlighted by the pandemic.

One such example is under the theme of accessible information. Despite the presence of R.A. 7277 and 11106 there are still noticeable shortcomings on this matter as revealed by the need for private volunteer groups to fill the information gap for the hard of hearing community. The situation was acknowledged by the House of Representatives when they called for a comprehensive report on the monitoring and implementation of RA No. 11106 and for an investigation on its delay.^18^ The deaf and mute also face additional challenges such as the inability to report verbally to emergency hotlines.^19^ This leads to difficulty in accessing timely healthcare services and must be addressed by providing alternative means of communication.

Access to healthcare services is one of the most important needs of PWDs, more so in this pandemic since their existing comorbidities place them at greater risk of infection and complications from COVID-19. In a country wherein private out-of-pocket payments continue to be the main form of accessing healthcare, financial resources would be easily drained should they get infected. In order to offset some of the costs, PhilHealth, the nation’s health insurance program, included hospitalization and testing for COVID-19 under their list of benefit packages. PWD livelihood programs by different departments of the government also provide healthcare services, together with skills and entrepreneurial training, and financial aid.

Although these programs are greatly beneficial, they are only able to provide for a small number of PWDs and require certain conditions to be met. One example is the KaGabay program of the Department of Labor and Employment which provided livelihood and financial aid during the pandemic to 49 beneficiaries. This program only included persons with work related disabilities who have lost employment due to work related sickness or injury.^20^ Several government programs also require PWDs to register and require proof of disability to be considered a beneficiary. However, these mechanisms are nonuniform, ambiguous, and pose several challenges, causing several PWDs to be overlooked.^21^ The Department of Social Welfare and Development Social Amelioration Program, a key response of the government to financial difficulties brought about by the pandemic, is another example of this unequal distribution. Local governments were tasked to deliver this cash assistance, but due to vague implementing guidelines and requirements, several members of the vulnerable population remain unassisted. Countless PWDs reported inability to receive cash aid due to reasons such as being unregistered PWDs, non-inclusion to beneficiary lists, inability to produce documents which are hard to acquire during the crisis, and even lack of available forms to fill up.^22^

Apart from these shortcomings, it was notable that some of these policies treated PWDs as a homogenous group without considering their specific needs and barriers to accessibility. This was seen in the Department of Interior and Local Government’s method of distributing food packs, vitamins, and medicine. Because each PWD experiences different barriers due to their unique impairments and functional limitations, the PWD community should be viewed as composed of several subsets. For these reasons, it is important to include PWDs and their representatives in the development of policies.

## Conclusion

This study has revealed the responsiveness of the Philippine government to include PWDs in their COVID-19 response. The presence of several policy instruments has proved the state’s cognizance for PWDs. This was exemplified by the advisory series released by the Philippine Commission on Human Rights which provided extensive guidelines on the rights of PWDs and the standards to uphold for them amidst the pandemic. Despite this, the study demonstrated that implementation remains to be a problem, and because of this impact from these policies has yet to be seen. There is a tendency for these policies and advisories to overgeneralize PWDs. It is important to recognize the heterogeneity of PWDs to better account for their specific needs. Moreover, there is not enough focus on adequate implementation and on evaluation of existing policies and programs. It is important to identify existing gaps and barriers to address these shortcomings and to be able to cover as many PWDs as possible. A comprehensive national database should be instituted to centralize registration across all local government units. This will also assist in creation of policies and programs that are suited to the PWDs needs. Efforts should be made to inform, guide, and educate PWDs of their rights and of existing programs. Most importantly, PWDs should be included in the discourse and decision-making process for policies that concern them to ensure programs developed are acceptable and accessible.

## Funding

No funding received from any institution or department.

## Competing interests

The authors completed the ICMJE Unified Competing Interest form (available upon request from the corresponding author) and declare no conflicts of interest.

## Ethical approval

This study did not use data collected from human subjects; informed consent was not required.

## Authors’ contributions

JVV, JCO, and DELP conceived the idea. JVV, JCO, AP, HKA, and PMV wrote the manuscript, collected data and literature with joint equal contribution by DELP. DELP assisted with article interpretation, supervised the development of work, helped in data interpretation and manuscript evaluation. All the authors read and approved the final manuscript.
